# Individual differences in co-representation in three monkey species (*Callithrix jacchus, Sapajus apella* and *Macaca tonkeana*) in the joint Simon task: the role of social factors and inhibitory control

**DOI:** 10.1007/s10071-022-01622-8

**Published:** 2022-05-05

**Authors:** Fabia M. Miss, Baptiste Sadoughi, Hélène Meunier, Judith M. Burkart

**Affiliations:** 1 Department of Anthropology, University of Zurich, 8057 Zurich, Switzerland; 2 Centre de Primatologie de l’Université de Strasbourg, Niederhausbergen, France; 3 Department of Life Sciences, University of Roehampton, London, UK; 4 Oniris-Nantes Atlantic College of Veterinary Medicine, Food Science and Engineering, Nantes, France; 5 Department of Behavioral Ecology, Johann-Friedrich-Blumenbach Institute for Zoology and Anthropology, University of Göttingen, Göttingen, Germany; 6 Leibniz Science Campus Primate Cognition, German Primate Center, Göttingen, Germany; 7 Laboratoire de Neurosciences Cognitives et Adaptatives, UMR 7364, Strasbourg University, Strasbourg, France

**Keywords:** Nonhuman primates, Joint action, Joint Simon task, Social bond strength, Inhibitory control, Social cognition

## Abstract

Behavioral coordination is involved in many forms of primate interactions. Co-representation is the simultaneous mental representation of one’s own and the partner’s task and actions. It often underlies behavioral coordination and cooperation success. In humans, the dyadic social context can modulate co-representation. Here, we first investigated whether individual differences in co-representation in the joint Simon task in capuchin monkeys and Tonkean macaques can be explained by social factors, namely dyadic grooming and sociality index, rank difference and eigenvector centrality. These factors did not predict variation in co-representation. However, in this specific task, co-representation reduces rather than facilitates joint performance. Automatic co-representation therefore needs to be inhibited or suppressed to maximize cooperation success. We therefore also investigated whether general inhibitory control (detour-reaching) would predict co-representation in the joint Simon task in Tonkean macaques, brown capuchin and marmoset monkeys. Inhibitory control did neither explain individual differences nor species differences, since marmosets were most successful in their joint performance despite scoring lowest on inhibitory control. These results suggest that the animals’ ability to resolve conflicts between self and other representation to increase cooperation success in this task is gradually learned due to frequent exposure during shared infant care, rather than determined by strong general inhibitory control. Further, we conclude that the joint Simon task, while useful to detect co-representation non-invasively, is less suitable for identifying the factors explaining individual differences and thus a more fruitful approach to identify these factors is to design tasks in which co-representation favors, rather than hinders cooperation success.

**Supplementary Information:**

The online version contains supplementary material available at 10.1007/s10071-022-01622-8.

## Introduction

Various natural forms of human and nonhuman primate cooperation like coalition formation, food sharing, group hunting, territorial defense, biparental care, communication or social play involve behavioral coordination with other individuals (Burkart et al. 2022; Clark 2006; Hrdy 2009; de Waal and Suchak 2010; Tomasello 2019; Heesen et al. 2021). Fine-tuned motor coordination can be enabled through the mental representation of not only one’s own but also the partner’s task and actions (Sebanz et al. 2003, 2005; Vesper et al. 2010, 2017). Co-representation involves basic perception–action matching (ideomotor theory or common coding, Prinz 1997; Hommel et al. 2001) and action simulation (simulation theory, Gallese and Goldman 1998) when individuals internally simulate actions performed by the co-actor and integrate this simulation with representations of own action goals and planned subsequent actions (Sebanz et al. 2007; Bekkering et al. 2009; Knoblich et al. 2011). As a part of self–other (SO) integration, this co-representation presumably facilitates joint performance because it allows for immediate predictions of the partners’ behaviors and enables individuals to prepare their actions in anticipation of their partner’s actions, and therefore refines motor coordination (Sebanz et al. 2006a; Sommerville and Decety 2006; Vesper et al. 2010; Butterfill 2012; Ruissen and de Bruijn 2016). Co-representation is involved in basic dyadic motor coordination requiring complementary actions, such as the joint Simon task (in humans: Sebanz et al. 2003; Ruys and Aarts 2010; in primates [platyrrhine and catarrhine monkeys]: Miss et al. 2022; Miss and Burkart 2018) or joint music performance (Novembre et al. 2016; see also vocal turn-taking in cooperative communication in callitrichids: Takahashi et al. 2013). Co-representation is particularly useful when individuals perform identical actions simultaneously and successful coordination increases with motor alignment through self-other (SO) integration, such as in joint grasping/lifting/pulling tasks or synchronization tasks (Newman-Norlund et al. 2008; Vesper et al. 2013, 2014; Meyer et al. 2016; Schmitz et al. 2017; in primates [chimpanzees]: Melis et al. 2006; Constable et al. 2021).

In human adults, some evidence suggests that social factors can modulate co-representation. For instance, co-representation emerged only with a friendly acting, supportive partner but not with an intimidating, competitive partner (Hommel et al. 2009), and between in-group (i.e., partners belonging to the same group) but not out-group members in case of a salient group categorization (McClung et al. 2013; see Iani et al. 2011 for no modulation with a minimal group categorization). Likewise, a competitive rather than cooperative set-up between the co-actors either in the joint Simon task (Iani et al. 2011) or in an unrelated dyadic task preceding the joint Simon task (Iani et al. 2014; Ruissen and de Bruijn 2016) hindered the emergence of co-representation. Moreover, co-representation increased with perceived inter-personal closeness between partners (assessed with the Inclusion of the Other in the Self scale, Shafaei et al. 2020). Intriguingly, co-representation even emerged with an invisible co-actor, if the partner believed that the complementary motor responses came from a social, intentionally acting partner instead of being automatically generated by an algorithm (Tsai et al. 2008; Sahaï et al. 2019). A first goal of our study was therefore to investigate whether and how social variables predict the co-representation previously reported in dyads of common marmosets (*Callithrix jacchus*), capuchin monkeys (*Sapajus apella*) and Tonkean macaques (*Macaca tonkeana*) tested with a joint Simon task (Miss et al. 2022; Miss and Burkart 2018).

The smooth motor coordination between individuals does not merely depend on strong SO integration, but also requires an optimal balancing between SO integration vs. SO distinction (e.g., Steinbeis 2016). Studies on neural inter-brain underpinnings of social interaction during real-time joint action tasks showed that co-actors’ brains are linked through coupled neural oscillations, for instance, during joint rhythmic behavior, joint speech, or joint movement behavior (Keller et al. 2014; Novembre et al. 2016; Djalovski et al. 2021). In particular, these inter-brain neural processes appear to play a crucial role in the regulation between SO integration (and social alignment) and SO distinction (Novembre et al. 2016; Gvirts and Perlmutter 2020; de Hamilton 2021). This distinction is most necessary in contexts when co-representation hinders rather than facilitates joint performance and thus cooperation success, as for instance, in joint interference tasks like the joint Simon task (Sebanz et al. 2003) and the joint flanker task (Atmaca et al. 2011), or imitation-inhibition tasks (Spengler et al. 2010), or perspective-taking tasks (Samson et al. 2010). Since co-representation and SO integration most likely emerge as an automatic process in these contexts (Decety and Sommerville 2003; Brass et al. 2009; see also Southgate 2020; Sebanz and Knoblich 2021), SO distinction requires active inhibition and suppression of co-representation. Our second goal of this study was therefore to investigate how primate co-representation in the joint Simon task is linked to individual differences in independently assessed inhibitory control.

The joint Simon task (Sebanz et al. 2003) is a basic dyadic motor coordination task where co-representation hinders joint performance and cooperation success. It has the advantage of making co-representation visible and thus allowing a non-invasive, behavior-based quantification of co-representation (e.g. in nonhuman primates, Miss et al. 2022; Miss and Burkart 2018). However, it is a joint interference task and therefore integrating the co-actor’s action and task requirements makes the distinction between one’s own and the partner’s task affordances more difficult. Therefore, joint performance success in this task crucially requires SO distinction.

The primate task design (Miss and Burkart 2018) adopted the auditory version of the joint Simon task from human studies (Sebanz et al. 2003; Ruys and Aarts 2010). In this experimental paradigm, two individuals share the task to correctly react to an external sound stimulus by choosing one of two response options, each individual being responsible for one of them. In particular, two different sounds require either answering on the left-hand or on the right-hand side of a response device (e.g. sound *A* requires answering on the *left-hand* side and sound *B* requires answering on the *right-hand* side) and in case of a correct choice, both partners receive a reward, independent of which actor provided it. This task set-up is similar to the cooperative designs used with human adults, in which dyads are *explicitly instructed* to cooperate, sometimes emphasized by the promise that the best performing dyads in a given group of participants will receive a reward (e.g., Tsai et al. 2008; Ruys and Aarts 2010; Iani et al. 2014).

The difficulty of the joint Simon task lies in the directional properties of the sounds, namely, the sounds are broadcast from either the left-hand or the right-hand direction, which creates either compatible trials (i.e., the side of the broadcast and the answer match) or incompatible trials (i.e., the side of the broadcast is opposite to the answer side). Typically, answering to incompatible stimuli is more difficult than answering to compatible ones when individuals share the task with a joint action partner (i.e., when one individual is responsible for answering on the left-hand side and the other one for answering on the right-hand side): *the joint Simon effect* (Sebanz et al. 2003; Ruys and Aarts 2010). Intriguingly, this Simon effect disappears when they do the exact same half part of the task on their own during an individual control condition (e.g., only answering to sound *A* when situated on the left-hand side). Thus, when sharing the task with a partner, typically error rates in response choices (mainly in primates) and in first heading directions (i.e., the first subtle movement or body orientation toward a response side, only measured in primates: Miss et al. 2022), and delays in reaction times (mainly in humans) are increased in incompatible trials (i.e., the joint Simon effect indicative of co-representation: Sebanz et al. 2003). To capture subtle differences in reaction times, animals would have to be trained to respond as quickly as possible. Instead, we found that errors in response choices as well as first heading directions were reliable variables to quantify co-representation in the joint Simon task in primates (Miss et al. 2022; Miss and Burkart 2018).

Since in the joint Simon task co-representation reduces rather than facilitates cooperation success (i.e., the total amount of rewards received by both), the amount of correct response choices (i.e., the cooperation success) in incompatible trials indicates how flexible co-representation is deployed to increase SO distinction and improve joint performance. Such flexibility requires the inhibition of co-representation, and electrophysiological and fMRI evidence indeed shows the recruitment of control mechanisms in the joint Simon task to inhibit motor responses when it is the partner’s turn, and to increase action monitoring and joint attentional processes (Sebanz et al. 2006b, 2007; Tsai et al. 2006; Ruissen and de Bruijn 2015). The latter is in line with the behavioral studies in primates showing a higher frequency of visual monitoring behavior directed at the partner when both individuals are engaged together in the task than when the partner is present but cannot engage in the task (i.e., blocked access to the response device; Miss et al. 2022; Miss and Burkart 2018).

Recent studies have addressed the evolutionary origin of co-representation and reported co-representation assessed with the joint Simon task in three primate species, the highly cooperative common marmosets, the intermediate brown capuchins, and the Tonkean macaques who less often engage in cooperative actions with each other (Miss et al. 2022). Common marmosets, like humans, qualify as cooperative breeders: in addition to mothers, other group members regularly contribute to rearing offspring, which increases infant growth and survival (Burkart et al. 2009; Hrdy 2009; Erb and Porter 2017). Capuchin monkeys and Tonkean macaques, in contrast, are independent breeders and the prevalence of cooperation among group members during everyday interactions is thus comparatively lower than in marmoset monkeys (Petit et al. 1992; Perry and Rose 1994; Thierry et al. 1994; Mendres and de Waal 2000; Bergstrom and Fedigan 2010; Burkart et al. 2014). Co-representation became weaker as general cooperativeness in a species increased, from Tonkean macaques, to capuchins, to marmosets. Thus, co-representation emerges in experimental contexts even in species such as the Tonkean macaque, in which dyads who are tolerant enough to engage in such a task together repeatedly are most likely less common than, for instance, in marmosets (Petit et al. 1992; Burkart et al. 2014; Martin et al. 2021). Moreover, the documented interspecific variation suggests that the more a species engages in cooperation (facilitated in particular by shared infant care among group members), the better it is to selectively suppress spontaneous co-representation if necessary (as in the joint Simon task), and therefore achieves higher cooperation success. It thus appears that primates (at least haplorrhines) generally co-represent their partners’ tasks and actions when engaged in a joint action task, but the flexibility to regulate co-representation and suppress it if this optimizes cooperation success appears higher in species who most routinely engage in joint activities during everyday life, rather than in bigger-brained species (Miss et al. 2022).

The suppression of co-representation may entirely depend on strong general inhibitory control abilities. In line with this, a study with 4–5-year-old children found that besides Theory of Mind (ToM), *stronger* motor inhibitory control skills (independently assessed with a modified day-night Stroop task, Gerstadt et al. 1994, and the pictures task, Burns et al. 2012) were associated with *weaker* co-representation in a joint action task requiring complementary actions (Milward et al. 2017). However, an exclusive reliance on general inhibitory control is not in line with the finding that among the three primate test species, brain size increases from marmosets to capuchins to Tonkean macaques (Deaner et al. 2007) and brain size and general inhibitory control tend to be correlated in primates (MacLean et al. 2014). Yet, the inhibition of co-representation was strongest in the marmosets, the species with the smallest brain.

An alternative to general inhibitory control is that the suppression of co-representation is achieved through repeatedly and frequently experiencing SO integration—distinction conflicts during joint action (*cooperative flexibility hypothesis,* Miss et al. 2022). Species in which group members are highly interdependent (de Oliveira Terceiro et al. 2021) and regularly engage in cooperative activities, particularly required for joint infant care (e.g., food sharing, coordination of infant transfers, group defense and vigilance or communicative exchanges, Burkart et al. 2022; Snowdon 2001; Takahashi et al. 2013; Guerreiro Martins et al. 2019; Hrdy and Burkart 2020) are likely to have recurrent opportunities to engage in joint actions and learn the necessary skills from an early age on. This may include practicing and learning to optimally balance SO integration and SO distinction during joint action and thus to selectively suppress automatic co-representation, for instance when coordinating complementary actions (e.g., handing over an infant from one carrier to another) or mutually exclusive activities among group members (e.g., feeding vs. vigilance). A training study with human subjects supports this alternative that inhibition of co-representation may be achieved independently of general motor inhibitory skills. Using a perspective-taking task (i.e., the Director’s task), Santiesteban et al. (2012) found that the prior training to inhibit imitation, but not of motor inhibitory control in general (assessed with a Stroop-like paradigm), increased the participants’ subsequent task success.

Our goal was to investigate if social factors or general inhibitory control ability could explain individual differences in co-representation in the Tonkean macaques, the brown capuchin and the common marmoset monkeys from Miss et al. (2022) and Miss and Burkart (2018). To examine the role of social factors, we collected from the Tonkean macaques [TM] and the brown capuchins [BC] observational data on social behaviors to quantify dyadic bond strengths (i.e., a dyadic grooming index (DGI) and a dyadic composite sociality index (DSI) [TM]) (Silk 2007), social rank differences (Elo-ratings) [TM, BC] (Neumann et al. 2011) and eigenvector centrality values based on the social affiliative network [TM] (Brent 2015). Individuals with high centrality values tend to have a large number of partners and more frequent affiliative interactions (Silk 2007; Cheney et al. 2016). In animals, particularly those which frequently cooperate in the wild, such as ravens, *Corvus corax* (Massen et al. 2015; Asakawa-Haas et al. 2016) or wolves, *Canis lupus* (Dale et al. 2020), dyads with stronger social bonds and closer in rank (but see Molesti and Majolo 2016 in barbary macaques, *Macaca sylvanus*) tend to be better cooperators in joint pulling tasks requiring simultaneous actions. Based on the findings in animals and humans, strong social bonds can be predicted to lead to stronger SO integration and co-representation. However, based on the conflicting role of co-representation and the necessity of SO distinction in the joint Simon task, we may likewise predict higher cooperation success (and thus *weaker* co-representation) in dyads with stronger social bonds. This is particularly likely if the suppression of co-representation does not require general inhibitory control ability but can be achieved otherwise, for instance through repeated exposure to SO conflicts in joint action contexts and training. This latter pathway would be consistent with the cooperative flexibility hypothesis, which proposes co-representation as a universal, automatic process in primates, and that the flexibility to suppress it is not primarily dependent on advanced general cognition but is enhanced when individuals are frequently exposed to SO integration—distinction conflicts.

To examine the role of general inhibitory control ability in suppressing co-representation in the joint Simon task, we tested in the Tonkean macaques, the brown capuchins and the common marmosets whether inhibitory control skills independently assessed with a detour-reaching task (MacLean et al. 2014; Schubiger et al. 2019; Gokcekus 2020) would predict individual differences in co-representation. Detour-reaching paradigms are frequently used to test the ability to inhibit or withhold pre-potent motor responses of directly reaching for an immediate apparent reward and instead make a detour (Amici et al. 2008; Vlamings et al. 2010; Manrique et al. 2013; Kabadayi et al. 2018). Since Tonkean macaques showed rather strong co-representation compared to much smaller brained common marmosets, and based on findings in humans (Santiesteban et al. 2012), we did not expect that general motor inhibition ability would explain variation in the strength of co-representation in these monkeys. Indeed, the species differences (Miss et al. 2022) suggest that stronger general inhibitory control might not primarily predict the ability to selectively suppress co-representation to improve cooperation success in the joint Simon task, whereas the habitual engagement in joint activities (cooperative flexibility hypothesis, Miss et al. 2022) does.

## Methods

### Strength of co-representation

**Fig. 1:**
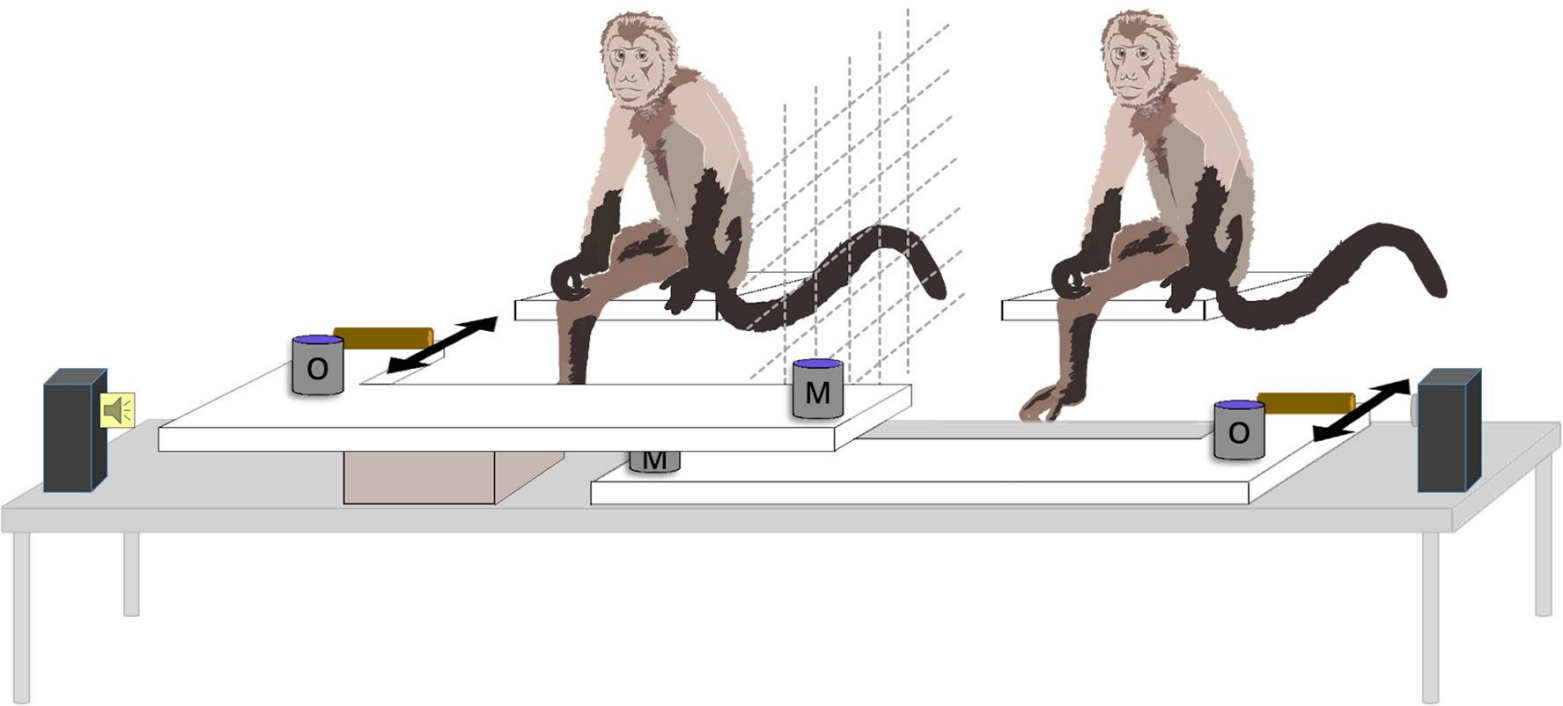
Experimental set-up describing the joint Simon task condition. The apparatus consisted of two sliding drawers with fixed handles to pull the drawers within reach and retrieve a food reward out of the cups in case of a correct choice. The focal individual could retrieve its food reward out of the outer cup (O) and the partner monkey out of the cup in the middle (M). In every trial, one of the two auditory stimuli “A” or “B” were broadcast from one of the two lateral speakers. Sound “A” asked for pulling the drawer on the left-hand side, whereas sound “B” asked for pulling the drawer on the right-hand side. The set-up was the same in all three test species

Data on individual differences in co-representation during the joint task were taken from an auditory version of the joint Simon task and available from nine marmosets, six capuchins and seven macaques (Miss et al. 2022; Miss and Burkart 2018). The same response device (adjusted to body size), training and testing procedure and test criteria were applied in the three species. The response device consisted of two sliding drawers, which could be pulled within reach with fixed handles, and contained two fixed cups each (one outer cup for the focal individual answering to the stimulus and one cup in the middle for the partner individual). The monkeys could swing the cups open to retrieve a food reward simultaneously in case of a correct choice (Fig. 1). The two drawers were connected with a cord going around a pole in the back. This mechanism moved the second drawer backwards and out of reach for the partner monkey as soon as one drawer was pulled. In the training phase the subjects were alone and the two sounds were broadcast from the middle (thus not creating any stimulus incompatibility), and the monkeys learned the association between the sound stimulus and the corresponding response side (the left-hand or the right-hand drawer). The individual learning criterion was to reach at least six sessions consisting of 12 trials with at least 75% correct choices. After subsequently passing the criterion of 75% correct choices in a joint task set-up, the individuals were then tested in the joint task condition in five sessions (respectively four in case of the two marmoset breeding pairs) consisting of 12 trials each. Differences across individuals in previous participation in cognitive tasks and in motivation were thus controlled to some degree by the requirement of passing several criteria (see also below), in particular to have learned the association between the sounds and sides. This precondition ensured that all individuals were motivated to participate in the tests and sufficiently attentive to the stimuli to learn the association and the task demands.

The training and testing procedure were identical. A separation grid was placed between the individual and its partner, allowing constant visual contact of the monkeys with each other. A screen permitted baiting of the cups out of sight of the monkeys and ensured that the drawers remained out of reach before the start of a trial. While attracting the subjects’ attention to the middle in front of the testing device, a trial started with the broadcast of the sound. Simultaneously, the screen was lifted and the two drawers were pushed within reach of the subjects. A trial ended when one of the two subjects pulled one of the two drawers, thus requiring an exclusive choice in each trial. In case of a correct choice, the subjects could retrieve the reward simultaneously from the outer and middle cup, respectively (i.e., joint reward) and consume it, while the other two cups were opened to uncover the non-baited side. In case of an incorrect choice, the non-selected cups were opened to uncover the baited side and the screen was lowered.

A test session started with two to six motivation pre-trials in which the sounds were broadcast from a central position (identical to the training phase) to ensure that the subjects remembered the corresponding sound-side association and to check their motivation of receiving food items as rewards. The test session was only started on a particular day if they chose each baited side twice correctly during these pre-trials. If this criterion was not met, the subjects were tested the following day. In every test trial, the sounds were played back from either the left- or the right-hand side, thus either eliciting stimulus incompatibility or not. In compatible trials, sound “A” was played back from the left-hand side and sound “B” from the right-hand side, whereas in incompatible trials, sound “A” was played back from the right-hand side and sound “B” from the left-hand side. The order of the two sounds and of the sides from which they were broadcast as well as the sides of the subjects were pseudo-randomized and counterbalanced. Since in the joint task, the individuals shared the task with a partner monkey, we expected a joint Simon effect (i.e., co-representation) in the focal individual, expressed in increased error rates in incompatible trials and consequently fewer joint rewards.

In the marmosets, the dyads were formed within their family groups (or consisted of the breeding pair, respectively) and preselected according to observations of affiliative behaviors during the training phase, such as entering together and remaining in proximity in the testing cage, participating next to each other in trials and occasionally food sharing. The composition of the dyads was the same over all sessions. In the brown capuchins and the Tonkean macaques, the dyads were not predefined but formed in every session according to which animals decided to enter the experimental facility together at the same time. According to this free partner choice method, the composition of the dyads could vary across sessions but the dyads contained only partners that tolerated each other’s close proximity in the context of receiving food simultaneously. In both species, this resulted in pairings with the same partner individual in at least two sessions (four individuals in the capuchin group even had the same partner in four or all sessions). Table 1 shows a description of all tested individuals.

**Table 1:** Description of the individuals of the three species tested with the joint Simon task

Subject	Sex	Age	Social status (A); Rank (B), (C)	Family group or breeding pair (A); Matriline (B), (C)	Partner individuals [session number]
(A) Common marmosets					
Jojoba	♀	9	Breeder	a	Marvin _[1–5]_
Marvin	♂	8	Breeder	a	Jojoba _[1–5]_
Jupie	♀	6	Helper	a	Jet _[1–5]_
Jet	♂	6	Helper	a	Jupie _[1–5]_
Joyce	♀	2	Helper	a	James _[1–5]_
James	♂	2	Helper	a	Joyce _[1–5]_
Lea	♀	8	Breeder	b	Kyros _[1–4]_
Kyros	♂	7	Breeder	b	Lea _[1–4]_
Jaja	♀	6	Breeder	c	Membo _[1–4]_
(B) Brown capuchins					
Willow	♀	12	3	Kolette	Balin _[1, 2, 3, 5]_, Capuche _[4]_
Koli	♀	6.5	4	Kolette	Bombers _[1, 3]_, Capuche _[2, 4, 5]_
Bombers	♂	5	6	Kolette	Conan _[1, 3, 5]_, Koli _[2, 4]_
Capuche	♀	4	9	Kolette	Willow _[1, 2, 4, 5]_, Balin _[3]_
Balin	♂	5	2	Willow	Willow _[1–5]_
Conan	♂	3.5	7	Willow	Bombers _[1–5]_
(C) Tonkean macaques					
Yannick	♂	9	11	Lady	Olaf _[1,2]_, Abricot _[3]_, Nereis _[4]_, Olli _[5]_
Nereis	♀	19.5	3	Nereis	Olli _[1,5]_, Olaf _[2]_, Anubis _[3]_, Nema _[4]_
Nema	♀	7.5	10	Nereis	Anubis _[1, 3]_, Nereis _[2]_, Olaf _[4, 5]_
Anubis	♂	5	15	Nereis	Abricot _[1, 4]_, Nereis _[2]_, Nema _[3, 5]_
Olli	♂	8	4	Olga	Nereis _[1, 4]_, Olaf _[2, 3, 5]_
Olaf	♂	6.5	12	Olga	Yannick _[1, 3]_, Nereis _[2]_, Olli _[4, 5]_
Abricot	♂	5	17	Olga	Anubis _[1, 3]_, Yannick _[2]_, Olaf _[4]_, Nereis _[5]_

Sex (♀ = female, ♂ = male), approximate age in years, social characteristics, and corresponding partner individuals listed according to the session number(s) in brackets

### Observations of social behaviors

#### Subjects

We collected behavioral data on 22 individuals (adults > 6 years, sub-adults > 4 years and < 6 years) in a group of semi-free ranging Tonkean macaques (*Macaca tonkeana*). The group consisted of 28 individuals during the period of data collection: 18 adults (9 females), four sub-adults (all males), five juveniles (< 4 years, too young to be reliably identified during the period of data collection) and one newborn. We also observed 14 individuals in a group of semi-free ranging brown capuchins (*Sapajus apella*) which consisted of 19 individuals during the study period: six adults (five females), six sub-adults (two females), five juveniles (three females) and two newborns. All animals were housed at the Primate Centre of the University of Strasbourg, France and were captive born. The Tonkean macaques and the capuchin monkeys both had permanent access to a wooded park of 3788 m^2^ and 2332 m^2^ respectively, connected to a heated indoor-outdoor shelter. They were provisioned with commercial primate pellets twice a day and with fresh fruit and vegetables once a week. Water was available ad libitum. The Tonkean macaques moreover had 24 h access to Machines for Automated Learning and Testing (MALT) with the option to perform several cognitive tasks (Fizet et al. 2017). The observations were conducted non-invasively and the study was performed according to the French legal requirements for the use of animals in research and complied with the EU Directive 2010/63/EU on the welfare of animals used for scientific purposes.

#### Data collection

To test for effects of the social relationships and dominance hierarchy on the joint Simon effect, we recorded affiliative and agonistic interactions in the Tonkean macaque group during focal samplings and ad libitum observations (Altmann 1974). Prior to the start of the observational data collection, the animals were habituated to the researcher’s presence inside their park for two weeks. Subsequently, behavioral data on the Tonkean macaque group were collected by two researchers (BS & FM) right before the start of the Simon task study (March 14 until July 5, 2018) and immediately after (November 13 until December 13, 2018), providing observational data from 5 months. This resulted in approximately 6.6 h of observation time per individual (mean ± SEM = 6.6 h ± 0.1 h), and 769 recorded conflictual events. A part of these data have already been used in another study (Ballesta et al. 2021).

All adult and sub-adult members of the group were studied with the focal sampling method during 10 min sessions. One adult male (‘Wotan’) was removed from the group on June 1, 2018 and transferred to another park. Therefore, the total amount of observational time was slightly lower (5 h) for this individual compared to the others. Observations were only recorded when the individual was in complete view (in the park or the outdoor shelter) and were balanced evenly throughout the day (8 h 30–18 h). An individual was observed only once a day and the order of the focal follows was assigned randomly every day. If the focal animal could not be located, the next assigned individual was observed instead. No more than four individuals from the assigned order could be skipped to avoid recording when most group members were in the indoor shelter or out of view. Focal samplings were only used for analyses if the total time during which the focal animal was in view was at least 5 min. Toward the end of the observational periods, specific individuals were prioritized to correct for unbalanced distribution of observational sessions across time.

Behaviors were defined based on the social repertoire of Tonkean macaques (Thierry et al. 1990, 2000). Dyadic affiliative interactions included social grooming (s) and social contact (s) defined as two individuals sitting next to each other with body contact. They were only recorded during the focal follows, while aggressive and submissive interactions were additionally recorded ad libitum. Agonistic interactions were described as physical conflicts (e.g., wrestles, bites, slaps), chases, threats (e.g., open-mouth threats, stamps, stares), and displacements (the arrival of an individual is followed by the immediate departure of the approached individual, e.g., from a resource such as food or a consorted female or MALT). Submissive behaviors in the context of agonistic interactions included flights, crouching, moving away, and screams, potentially combined with facial expressions (e.g., silent-bared teeth). The actor, receiver and possible retaliation were recorded for every agonistic interaction (see supplementary material on hierarchy analysis).

Behavioral observations were recorded either on paper or on an IPod Touch with the Animal Pro Behavior software (Newton-Fischer, University of Kent 2012). Based on an entire week of behavioral observations (total of 89 focal follows), inter-observer reliability between BS and FM was calculated on the durations (s) of observed social grooming and social contact behavior (ICC = 0.99) and on the recorded agonistic events as well as the identities of the observed individuals (Cohen’s *κ* = 0.89).

### Data analysis

#### Hierarchy analysis

All the statistical analyses were conducted in R (version 3.5.3). With the Tonkean macaque data, we calculated Elo-ratings (Neumann et al. 2011) and David’s scores (de Vries et al. 2006) with the package ‘EloRating’ and rank stabilities with the package ‘Perc’ (Fujii et al. 2019) from a sequence of agonistic interactions recorded during the observational periods (Vilette et al. 2020) (see supplementary material on hierarchy analysis, Table S1 and S2). We then used the ranking output based on the Elo-ratings to define the dominance hierarchy and entered the scores of the individual’s absolute difference in rank compared to its joint action partner as fixed factors in the model calculation.

We could not base the dominance ranking for the capuchin monkeys on quantified data due to a lack of available behavioral data within the composition of the group during the Simon task test period from Miss et al. (2022). Instead, two observers estimated the hierarchy through ad libitum observations of agonistic interactions describing dyadic (physical) aggression and submissive behavior in resource related and social contexts, such as spatial displacements, supplants, avoidances, flights and screams (Leca et al. 2002; Bergstrom and Fedigan 2010).

#### Dyadic grooming index, dyadic composite sociality index and social network analysis in the Tonkean macaques

We turned all behavioral data into rates and included only data of the adult and sub-adult group members in the analyses (*n* = 22). For the observed dyads in the joint task, we calculated dyadic grooming indices (DGI) and dyadic composite sociality indices (DSI) based on the durations of the behaviors *grooming* and *sitting in contact* (Silk et al. 2013). DGI_(*xy*)_ were calculated as the total grooming time of a dyad *xy* divided by the mean grooming time across all dyads. DSI_(*xy*)_ were calculated as follows:

$${\text{DSI}}\left( {xy} \right) = \frac{{\mathop \sum \nolimits_{i = 1}^{2} \frac{{f_{ixy} }}{{\overline{f}_{i} }}}}{2}$$

while ${f}_{ixy}$ is the total time of grooming or sitting in contact for dyad *xy* and ${\overline{f} }_{i}$ is the mean grooming time or the mean sitting in contact time across all dyads. Therefore, high values of the DGI and the DSI represent dyads that had more frequent and/ or longer lasting affiliative interactions than the average dyad in the group and low values represent dyads which had less frequent and/ or shorter affiliative interactions than the average dyad.

To analyze a potential link between the subjects’ positions in an affiliative network of the group and their joint Simon effects, we further used the durations of the grooming and social contact behaviors to calculate eigenvector centrality measures with social network analysis (Brent 2015) using the package “igraph” (supplementary material, Fig. S1).

#### Model calculation

We calculated binomial generalized linear mixed effect models (glmm) using the package “lme4” with the Tonkean macaque and brown capuchin data to analyze a potential influence of social factors on the subjects’ strength of co-representation (i.e., joint Simon effect). Accordingly, to test for a potential influence of social factors on the Tonkean macaques’ joint Simon effects, we built a model on the joint task data with choice (either correct or incorrect answer) as a binary response variable and compatibility, absolute rank difference, DGI, DSI and eigenvector centrality as fixed factors. To test for a potential influence of social rank differences on the capuchins’ joint Simon effect, we built a model on the joint task data with choice as a binary response variable and compatibility and absolute rank difference as fixed factors. Both models included individual, session and partner as random factors, and were compared to a control model, containing only the control factor (compatibility) and the random factors. For all statistical analyses, model parameters were approximated using maximum likelihood estimation and model performance was assessed by likelihood ratio tests. All figures were generated using the package “ggplot2”.

### Inhibitory control

#### Subjects

In the detour-reaching task, we tested seven Tonkean macaques and five brown capuchins from the same study groups, and added the data of five common marmosets (*Callithrix jacchus*) tested with the identical detour-reaching task (Gokcekus 2020). All individuals also participated in the joint Simon task studies from Miss et al. (2022) and Miss and Burkart (2018). The marmosets were captive-born and housed in family groups at the Primate Station of the University of Zurich, Switzerland in heated indoor enclosures with access to outdoor enclosures during appropriate outdoor temperatures (> 10 °C). They were provisioned with mash and fresh fruit and vegetables every day. Water was available ad libitum. The research was approved by the Kantonales Veterinäramt, license number 223/16. For all study groups, subject participation was voluntary, the normal feeding routine was maintained during testing and animals were never food or water deprived.

#### Procedure

**Fig. 2:**
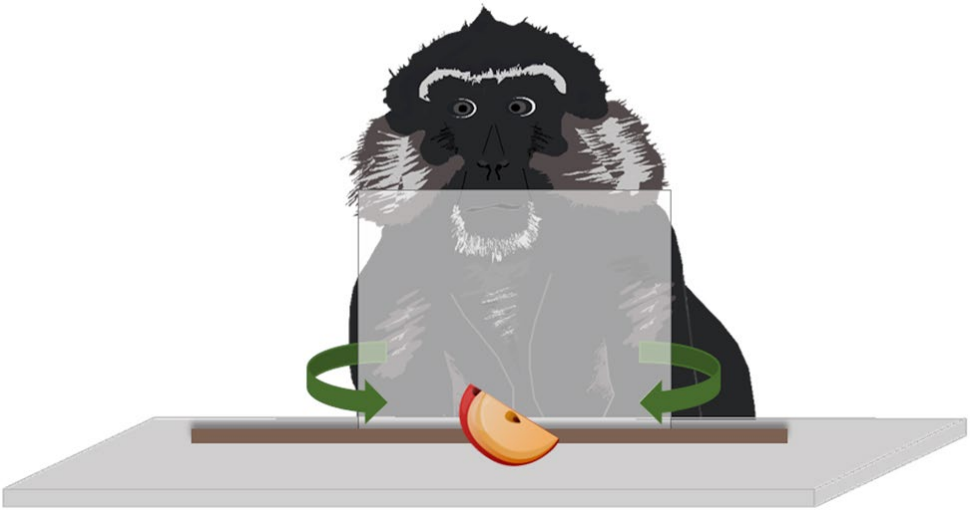
Experimental set-up in the detour-reaching task. To retrieve a food item placed behind a transparent Plexiglas panel, the subject had to reach around the panel. The set-up was the same in all three test species

We measured inhibitory control ability with a detour-reaching task adapted for primates (MacLean et al. 2014; Schubiger et al. 2019). The task required the individual to reach around a transparent barrier (Plexiglas panel: 25 cm × 25 cm for the Tonkean macaques, 18 cm × 18 cm for the capuchin monkeys and 8 cm × 8 cm for the marmoset monkeys) to retrieve a food item placed behind it (Fig. 2). The panel was vertically attached to the top of a wooden board. We conducted five sessions consisting of 12 trials (60 trials in total). In each session, the reward appeared four times in each of three possible locations (*central* = fully behind the panel; *left* = half exposed; and *right* = half exposed) in a counterbalanced and pseudo-randomized order with the rule that the reward never appeared in the same location in more than two consecutive trials. In the more difficult central trials, the reward was placed in the middle of the Plexiglas barrier so that it was fully occluded by it. The two lateral conditions served as a distraction and an attenuation of the level of difficulty since the subject could directly reach for the food item.

In a familiarization phase, every individual was given the opportunity to explore the Plexiglas panel through the grid for 10 min without a food reward present. In the test trials, we first placed a cardboard screen between the grid and the panel to occlude the positioning of the food item in one of the three locations (i.e., central, left or right). As soon as the individual was attentive, we called her name and removed the cardboard screen. In case the first attempt was not successful, we kept the panel in place for max. 2 min to allow further attempts to reach around the panel and retrieve the food item. The individuals completed a session on the same day in as many trial-blocks as needed. All trials were video-recorded.

### Data analysis

We scored all trials from the videos as either a successful first attempt (i.e., directly reaching around the panel) or not (i.e., reaching into the panel first) and calculated the measure of successful detour-reaching per individual as the percentage of correct trials at first attempt out of the 20 central trials. We assessed interrater reliability (Cohen’s *κ* = 0.97) for successful first attempts in the Tonkean macaques and the brown capuchins with 20% randomly selected video sessions. For the model calculations, we built glmms using the package “lme4” with the Tonkean macaque, the brown capuchin, and the marmoset data to analyze a potential influence of inhibitory control ability on the individuals’ strength of co-representation (i.e., joint Simon effect). Accordingly, we built models on the joint task data with choice as a binary response variable and compatibility and inhibitory control as fixed factors. The models included individual (nested in species in the analysis across the three species), session and partner as random factors, and were compared to a control model, containing only the control factor (compatibility) and the random factors.

For species comparisons in inhibitory control ability, we calculated a binomial glmm with the individual detour-reaching scores (either successful or failed first attempt) as binary response variable and all variables of interest (species, session, age, sex) as fixed factors. This model was compared to the null model consisting of the intercept and random effects only. Individual nested in species, and session were included as random factors. For the fixed factor session, we set a priori polynomial contrast to test for trends across time. For pairwise comparisons between species, we performed Tukey adjusted post hoc tests using the package “emmeans”. Variance Inflation Factors (VIFs) were calculated to examine if predictors did not violate any multicollinearity assumptions using the package “car” (all VIF scores < 2). The proportion of the total variance accounted for by the model was assessed by the conditional R^2^_GLMM_ value using the package “MuMIn”. For all statistical analyses, model parameters were approximated using maximum likelihood estimation and model performance was assessed by likelihood ratio tests. All figures were generated using the package “ggplot2”.

## Results

### An influence of social factors on the joint Simon effect?

We tested if social factors estimated with the social rank difference (Elo-ratings), the integration in the affiliative social network (eigenvector centrality values), and the bond strength with a given partner (the dyadic grooming index (DGI) and the dyadic composite sociality index (DSI)) could explain variation in co-representation in the Tonkean macaques and the brown capuchin monkeys. We built models on the joint Simon task data with choice as a binary response variable and found that the models did not improve compared to the control models containing only the fixed factor compatibility and the random factors [Tonkean macaques: $\chi_{4}^{2}$  = 2.64, *p* > 0.05, ∆AIC = 5.36, *N*_total_ = 209, *N*_individuals_ = 7; brown capuchins: $\chi_{1}^{2}$  = 0.13, *p* > 0.05, ∆AIC = 1.87, *N*_total_ = 184, *N*_individuals_ = 6]. In both models, only the fixed factor compatibility remained as a significant predictor, and none of the other fixed factors (absolute rank difference, eigenvector centrality, DGI, DSI) showed a significant effect (Table 2).

**Table 2:** The role of social factors in explaining variation in co-representation

Species	Fixed factor	*β*	SE	95% CI	OR	*z*	*p*
(a) Tonkean	Intercept	1.05	0.43				
macaques	Compatibility	− 2.75	0.35	− 3.43, − 2.07	0.06	− 7.87	*3.53* × *10*^–15^***
	Absolute rank difference	0.07	0.06	− 0.04, 0.19	1.07	1.22	0.22
	Eigenvector centrality	-0.32	0.59	− 1.47, 0.83	0.73	− 0.55	0.58
	DGI	0.09	0.20	− 0.29, 0.48	1.09	0.48	0.63
	DSI	− 0.01	0.10	− 0.20, 0.19	0.99	− 0.08	0.94
				*N* = 7 individuals, $\chi_{4}^{2}$ = 2.64, *p* = 0.62, ∆AIC = 5.36			
(b) Brown	Intercept	1.03	0.31				
capuchins	Compatibility	− 1.98	0.33	− 2.63, − 1.33	0.14	− 5.95	*2.66* × *10*^–9^***
	Absolute rank difference	0.03	0.08	− 0.12, 0.18	1.03	0.36	0.72
				*N* = 6 individuals, $\chi_{1}^{2}$ = 0.13, *p* = 0.72, ∆AIC = 1.87			

The effect of stimulus compatibility, absolute rank difference, eigenvector centrality (i.e. integration in the affiliative social network), DGI and DSI (i.e. bond strength) with a given partner on an individual’s strength of co-representation (i.e. joint Simon effect; errors in incompatible vs. compatible trials) in (a) the Tonkean macaques (*n* = 7) and (b) the brown capuchins (*n* = 6). Parameter estimates, standard errors, 95% confidence intervals (CI), odds ratios (OR) and statistical significance are obtained from generalized linear mixed effect models. Significant effects are indicated with *p*-values in italics

**Fig. 3:**
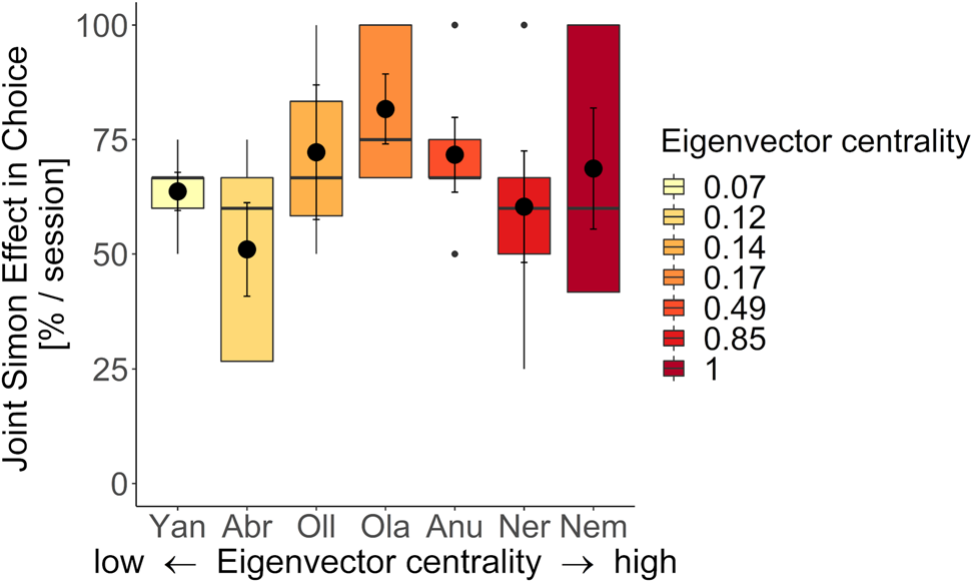
Relationship between the integration in the social network and co-representation. The strength of co-representation (i.e., joint Simon effect; % incorrect choices in incompatible minus compatible trials per session) in the Tonkean macaques is shown according to an individual’s eigenvector centrality value from low (light shading) to high (dark shading). The boxes and whiskers represent medians and lower and upper quartile scores. Error bars represent standard errors of the mean. More peripheral individuals in the affiliative social network and more central individuals did not differ in the strength of their co-representation (*p* > 0.05)

Therefore, individuals with partners of similar rank, or highly connected individuals with a large number of affiliative well-connected partners, or individuals with more strongly bonded partners generally did not show weaker or stronger co-representation (Table 2, Fig. 3 and supplementary material Fig. S2 & S3).

### Association between inhibition (detour-reaching) and the joint Simon effect?

**Fig. 4:**
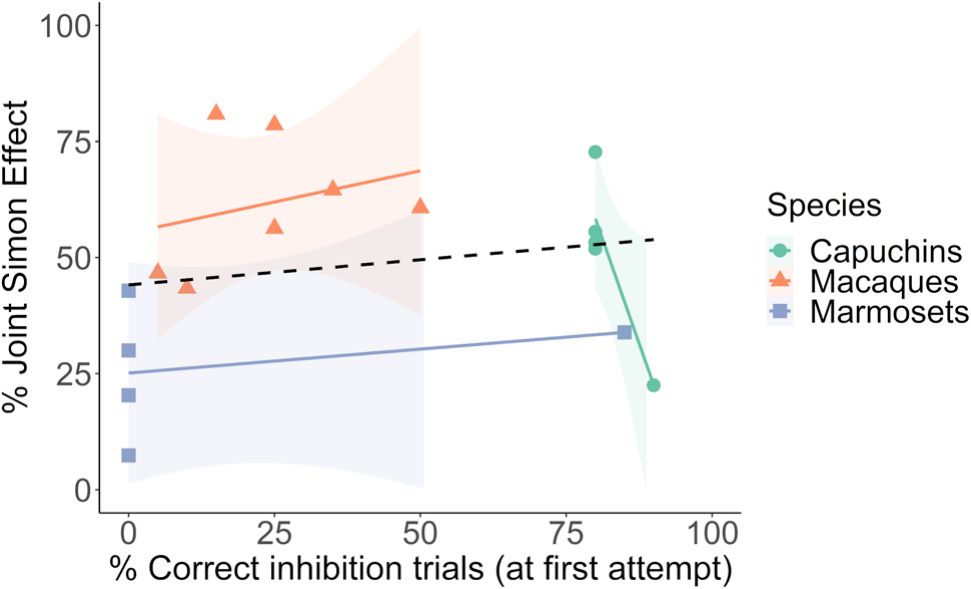
Relationship between inhibitory control ability and co-representation. Every individual’s joint Simon effect measure (i.e., the observed difference of incorrect choices between incompatible and compatible trials) is shown in relation to its inhibitory control measure (i.e., observed correct inhibition trials at first attempt in the detour-reaching task) in the Tonkean macaques (*n* = 7), the brown capuchins (*n* = 5), and the common marmosets (*n* = 5). The regression lines are the correlations between joint Simon effect measures and inhibitory control measures per species (solid) and overall (dashed) and do not show model predictions. The shaded areas display 95% confidence intervals

Better inhibitory control ability assessed with a detour-reaching task was not associated with weaker (or stronger) co-representation (i.e., smaller or larger joint Simon effect), neither when analyzed across all individuals [$\chi_{1}^{2}$  = 0.13, *p* > 0.05, ∆AIC = 1.87, *N*_total_ = 686, *N*_individuals_ = 17] nor when analyzed within each species (Table 3, Fig. 4).

**Table 3:** The role of inhibitory control ability in explaining variation in co-representation

Species	Fixed factor	*β*	SE	95% CI	OR	*z*	*p*
Overall	Intercept	1.10	0.17				
	Compatibility	− 1.75	0.17	− 2.09, − 1.42	0.17	− 10.37	< *2* × *10*^–16^***
	Inhibition	− 9.68 × 10^–4^	0.00	− 0.01, 0.00	1.00	− 0.37	0.71
			*N* = 17 individuals, $\chi_{1}^{2}$ = 0.13, *p* = 0.72, ∆AIC = 1.87				
Tonkean macaques	Intercept	1.36	0.36				
	Compatibility	− 2.70	0.34	− 3.37, − 2.03	0.07	− 7.89	*2.98* × *10*^–15^***
	Inhibition	− 7.28 × 10^–5^	0.01	− 0.02, 0.02	1.00	− 0.01	1.00
				*N* = 7 individuals, $\chi_{1}^{2}$ = 0.00, *p* = 1.00, ∆AIC = 2.00			
Brown capuchins	Intercept	3.49	3.68				
	Compatibility	− 2.15	0.38	− 2.90, − 1.41	0.12	− 5.66	*1.53* × *10*^–8^***
	Inhibition	− 0.03	0.04	− 0.11, 0.06	0.97	− 0.64	0.52
			*N* = 5 individuals, $\chi_{1}^{2}$ = 0.41, *p* = 0.52, ∆AIC = 1.59				
Common marmosets	Intercept	0.90	0.18				
	Compatibility	− 1.08	0.23	− 1.54, − 0.63	0.34	− 4.65	*3.32* × *10*^–6^***
	Inhibition	5.75 × 10^–4^	0.00	− 0.01, 0.01	1.00	0.17	0.86
			*N* = 5 individuals, $\chi_{1}^{2}$ = 0.03, *p* = 0.86, ∆AIC = 1.97				

The effect of inhibitory control ability assessed with a detour-reaching task on an individual’s strength of co-representation (i.e., joint Simon effect; errors in incompatible vs. compatible trials) overall across the three tested species (*n* = 17), and separately in the Tonkean macaques (*n* = 7), the brown capuchins (*n* = 5), and the common marmosets (*n* = 5). Parameter estimates, standard errors, 95% confidence intervals (CI), odds ratios (OR) and statistical significance are obtained from generalized linear mixed effect models. Significant effects are indicated with *p*-values in italics

When testing for species differences in inhibitory control ability, the full model explained the data better than the null model [$\chi_{8}^{2}$ = 21.48, *p* = 0.006, ∆AIC = 5.48, *N*_total_ = 340, *N*_individuals_ = 17] and revealed a significant effect of species and session (supplementary material, Table S3 and Fig. S4). The monkeys improved their inhibitory control score over time (liner trend of session: *β* (SE) = 1.61 (0.39), 95% CI = [0.84, 2.38], *z* = 4.10, *p* < 0.001). The capuchin monkeys generally had the highest inhibitory control scores (mean ± SEM = 82.00% ± 2.00%), followed by the Tonkean macaques (mean ± SEM = 23.57% ± 5.85%) and the common marmosets (mean ± SEM = 17.00% ± 17.00%). Pairwise Tukey adjusted post hoc tests revealed that the inhibitory control scores were significantly higher in the capuchins than in the marmosets (*β* (SE) = 5.11 (1.33), 95% CI = [1.99, 8.24], *z* = 3.83, *p* < 0.001) and higher than in the Tonkean macaques (*β* (SE) = 3.27 (1.01), 95% CI = [0.91, 5.63], *z* = 3.24, *p* = 0.003). No difference was found between the marmosets and the Tonkean macaques (*β* (SE) = − 1.85 (1.24), 95% CI = [− 4.75, 1.06], *z* =− 1.49, *p* = 0.30) (Fig. 4).

## Discussion

Individual variation in co-representation in the joint Simon task in Tonkean macaques, capuchin monkeys and common marmosets were not predicted by various social factors representing dyadic bond strength, social rank differences and connectedness in the affiliative social network, or general inhibitory control ability as measured with a detour-reaching task. Several explanations may account for these results, as we will discuss in detail below. First, the variation in social bond strength may have been too small in the primate data containing only tolerant dyads. Second, the specific affordances of the joint Simon task may have masked an effect of social bond strength. If a stronger bond between partners increased spontaneous co-representation, but also increased the effort put into maximizing cooperation success by suppressing co-representation, the net effect may not be visible because these opposing effects cancel each other out. This is particularly likely if general inhibitory control is not the main factor determining the strength of co-representation, which was indeed not the case in the primates tested here. The overall pattern thus appears most consistent with the cooperative flexibility hypothesis, namely that co-representation is an automatic default mechanism readily emerging when primates tolerantly engage in joint activities, but that the flexibility to balance SO integration vs. distinction requires practice and is therefore highest in those species routinely cooperating which is facilitated in particular by shared infant care.

A first explanation for the absence of a relationship between co-representation and social factors in the primates tested here may be that the variation in social bond strength was not large enough to detect any effect. To be able to conduct the joint Simon task at all in the capuchin monkeys and the Tonkean macaques, the dyads were formed in every session according to the animals coming voluntarily inside the experimental facility together at the same time. We therefore could only test highly tolerant dyads and lack strong social contrasts, such as co-representation in non-tolerant dyads, and dyads that are composed of different groups (in-group—out-group effects). In the marmosets, all dyads of a group are generally tolerant enough to be tested together, thus leading to ceiling effects regarding social tolerance. This first explanation is not unlikely because in humans, the influence of social factors on co-representation is often only observable in strong social contrasts. In particular, co-representation was observed in a cooperative but not a competitive task framing (Iani et al. 2011, 2014; Ruissen and de Bruijn 2016) and between in-group but not out-group members (Müller et al. 2011; McClung et al. 2013) but only if the group categorization was made highly salient (Iani et al. 2011). Further, it emerged only with a friendly acting, supportive partner but not with an intimidating, antagonistic partner (Hommel et al. 2009). It thus seems that task sharing and therefore co-representation at least in humans is maintained as long as the relationship between the interaction partners is “good enough”, i.e. is not negative. This emphasizes the necessity for future studies with nonhuman primates to prioritize finding ways to also successfully test dyads at the lower end of social tolerance and relationship quality.

However, the requirement of strong social contrasts to detect an influence of social factors on co-representation may come from the fact that in humans, co-representation in joint Simon tasks is typically much weaker than co-representation observed in primates, and social effects may therefore be more difficult to detect. In animals, experimental studies on coordination behavior in the joint string-pulling task yield ambiguous results concerning the association between cooperation success and affiliation or closeness in rank between joint action partners. A positive relationship between cooperation success and social bond strength, closeness in rank, but further also inter-individual tolerance was found in wolves and ravens, both species that frequently cooperate in the wild (Massen et al. 2015; Asakawa-Haas et al. 2016; Marshall-Pescini et al. 2017; Dale et al. 2020). On the contrary, in barbary macaques, pairs with one adult and one low ranking juvenile were the most successful cooperators (Molesti and Majolo 2016) and in chimpanzees, cooperation was more successful between kin and individuals close in rank rather than between more strongly bonded individuals (Suchak et al. 2014). Therefore, depending on the species, inter-individual social tolerance might be just as, or more important than affiliation for cooperation success in joint action tasks. In our joint Simon task study in a group of 28 Tonkean macaques (22 individuals were ranked), mostly individuals of intermediate rank (five individuals were placed between rank 10 and 17 in the dominance hierarchy and only two individuals were higher ranked) participated. Consequently, joint action partners were mostly close in rank. In the group of 19 brown capuchins (14 individuals were ranked), the dyads showed almost exclusively the same pair-constellation over all sessions, which were those closest in rank and/or maternal kin. The alpha male did not tolerate the partner individual to simultaneously retrieve a rewarding food item and could therefore not be tested in the joint task. This is consistent with a potentially lower tolerance level for access to food in the most dominant male toward younger adult males observed in groups of capuchin monkeys in the wild (Janson 1985). The narrow range of rank differences and some preference for kin between partners in both monkey groups indeed suggest that all dyads that participated in the joint Simon task were highly tolerant.

The experimental results of animal studies on the joint string-pulling task indicate that in a joint action task in which cooperation success increases with SO integration and co-representation, also rather small differences in social tolerance and bond strength can be reflected in different levels of dyadic cooperation. Thus, also in the joint Simon task in primates, dyads with stronger social bonds may show stronger co-representation but simultaneously, they might make a greater effort to increase cooperation success which requires SO distinction in this specific task. A second explanation for the absence of a relationship between social factors and co-representation in the primates tested here may thus be that more affiliative or closer ranked dyads are more inclined to spontaneously co-represent their partner’s task and actions, but they are also better at suppressing it to maximize cooperation success. In a group of only highly socially tolerant individuals, this may result in the absence of any observable relationship in the joint Simon task. This is particularly likely if the ability to suppress (or activate) co-representation is not entirely determined by strong general cognitive factors (i.e., executive functions such as inhibitory control ability, or ToM).

Indeed, we found that general motor inhibitory control as measured with a detour-reaching task was linked neither to co-representation nor the ability to suppress it, within each of the three species but also when all species were analyzed together. Among the three species, the common marmosets showed the lowest level of motor inhibitory control, as expected from the fact that they have by far the smallest brains among the primates tested here (Deaner et al. 2007; MacLean et al. 2014; Burkart et al. 2017). Nevertheless, they were the least affected by automatic co-representation and showed the highest cooperation success*.*

Depending on the set-up and task administration, detour-reaching paradigms may rely more or less on additional skills such as causal reasoning, rule learning, attention or visual acuity, which may favor the use of detour task batteries over single tasks for species comparisons (Kabadayi et al. 2018). Variation in task-specific factors across species was controlled to some degree in our design by applying the same set-up (size of the barrier adjusted to body size) and procedure (in particular familiarization phase and number of trials) in all monkeys tested here, but individual differences in prior experience with cognitive tasks existed. We only measured one type of inhibitory control (detour-reaching) and different inhibition tasks likely measure different traits of this general cognitive skill (Audet and Lefebvre 2017). These include for instance motor inhibition (e.g., detour-reaching tasks, MacLean et al. 2014), self-control (e.g., delayed reward tasks, Evans et al. 2012) or the use of alternative behavioral strategies (e.g., reversal learning tasks, Manrique and Call 2015, or set-shifting tasks, Shnitko et al. 2017). Importantly, inhibitory control ability appears highly dependent on the context (e.g., in primates: Amici et al. 2018, in dogs: Brucks et al. 2017) and some species, despite having strong general inhibitory control, may show less behavioral flexibility in situations that require for instance a flexible switching of strategy adjusted to the social context (Amici et al. 2018). Correspondingly, highly flexible behavior in a social context (such as regulating or suppressing co-representation when necessary to optimize cooperation success) may be present in highly social species showing limited general inhibitory control, such as the common marmosets.

Thus, the primate data suggest that rather than primarily relying on general inhibitory control, the ability to suppress co-representation when necessary may be linked to specific processes of SO distinction in the motor domain (see Santiesteban et al. 2012), which may be trained when repeatedly engaging in cooperative interactions that require the balancing between SO integration vs. SO distinction. Highly social and interdependent species who rely on cooperation in their everyday life to facilitate joint infant care taking, such as humans and common marmosets (Hrdy 2009; Erb and Porter 2017), have recurrent opportunities to accumulate greater experience in joint activities and to become competent cooperators (e.g., in cooperative problem solving, Martin et al. 2021). This may advance the acquisition of abilities in social learning, such as behavior copying and imitation (Fletcher et al. 2012, in marmosets: Voelkl and Huber 2000, 2007), communication (Goldstein and Schwade 2008, in marmosets: Gultekin and Hage 2017; Takahashi et al. 2017), or coordination of attention and action (Bakeman and Adamson 1984; Moll et al. 2008). The necessity to coordinate complementary motor actions, such as handing over infants from one carrier to another or turn-taking during antiphonal calling (Snowdon 2001; Takahashi et al. 2013), and mutually exclusive activities among group members (e.g., feeding vs. vigilance, Brügger et al. 2022) arises frequently during shared infant care (Burkart et al. 2022; Hrdy and Burkart 2020). Such joint endeavors crucially require an optimal balancing between SO integration and SO distinction. Thus, during development and from an early age on, through interactions with their mothers, other caregivers (allomothers) and peers, infants may learn continuously when to merge and when to dissociate themselves from the other, while general cognitive skills (executive functions such as inhibitory control, and ToM) develop in parallel and may, at an advanced stage, come to support this process. Thus, a cooperative lifestyle may enhance the selective cognitive mechanisms to regulate and suppress co-representation when necessary through exposure to SO integration—distinction conflicts in joint action contexts and practice. This pathway of a supportive but not primary involvement of higher order cognition is consistent with the finding that stronger general inhibition and ToM skills were associated with weaker co-representation in a joint action task requiring complementary actions in 4- to 5-year-old children (Milward et al. 2017). Note that in humans, co-representation is hardly ever visible in actual errors but only in marginal delays in reaction times (commonly ranging between 10 and 30 ms; e.g., Sebanz et al. 2003; Kiernan et al. 2012; Pfister et al. 2014).

In the future, it is paramount to find ways to quantify both processes separately, the tendency to merge (i.e., spontaneously co-represent) as well as the ability to enhance SO distinction (i.e., suppress co-representation), and to measure for each of them separately how they are modulated by social factors. Joint Simon task studies in humans suggest that the influence of social factors increases with mutual dependency between co-actors (e.g., Ruys and Aarts 2010; Ford and Aberdein 2015). For such future investigations, it is thus desirable to find joint action tasks in which cooperation success increases with SO integration and co-representation without SO distinction to get rid of the confounding effect of the necessity to suppress automatic co-representation. Such paradigms may favor tasks with identical simultaneous task roles and include for instance synchronous movement (Kirschner and Tomasello 2009; Valdesolo et al. 2010), action imitation (Brass and Heyes 2005), or the mental coordination of decision-making (McClung et al. 2017). In fact, distinguishing between coordination tasks in which co-representation facilitates *versus* reduces cooperation success may as well help to explain weak or ambiguous results often reported in human co-representation in the joint Simon task (e.g., Sebanz et al. 2003; Pfister et al. 2014).

In sum, the emerging studies investigating the evolutionary origin of co-representation clearly show that co-representation is not unique to humans but most likely ancestral in primates, or at least haplorrhines. The flexibility to suppress co-representation when necessary among primates appears not contingent on strong general motor inhibitory control (and advanced higher order cognition) but seems rather strongest in those species who most routinely rely on, and thus accumulate greater experience in cooperation during their daily life, such as the cooperatively breeding common marmosets and humans. The advanced cognitive abilities of humans compared to primates were therefore not a precondition for the emergence of co-representation and cooperative flexibility during evolutionary times. Rather, it allowed our ancestors to deploy preexisting cooperative predispositions in ever more complex ways.

## Supplementary Information

Below is the link to the electronic supplementary material.

Supplementary file1 (DOCX 734 kb)
