# Supplementary material for: Individual differences in co-representation in three monkey species (Callithrix jacchus, Sapajus apella and Macaca tonkeana) in the joint Simon task: the role of social factors and inhibitory control
Source: Anim Cogn. 2022 May 5;25(6):1399–415. doi: 10.1007/s10071-022-01622-8 (PMC9652238; doi:10.1007/s10071-022-01622-8)
Supplement: Supplementary file 1 — Supplementary file1 (DOCX 734 kb) [file 10071_2022_1622_MOESM1_ESM.docx]

Supplementary Material for

**Individual differences in co-representation in three monkey species (*Callithrix jacchus, Sapajus apella* and *Macaca tonkeana*) in the joint Simon task: The role of social factors and inhibitory control**

Animal Cognition

Fabia M. Miss^1,2*^, Baptiste Sadoughi^2,3,4,5,6^, Hélène Meunier^2,7^, Judith M. Burkart^1^

^1^ Department of Anthropology, University of Zurich, Switzerland

^2^ Centre de Primatologie de l’Université de Strasbourg, Niederhausbergen, France

^3^ Department of Life Sciences, University of Roehampton, London, United Kingdom

^4^ Oniris – Nantes Atlantic College of Veterinary Medicine, Food Science and Engineering, Nantes, France

^5^ Department of Behavioral Ecology, Johann-Friedrich-Blumenbach Institute for Zoology & Anthropology, University of Göttingen, Göttingen, Germany

^6^ Leibniz ScienceCampus Primate Cognition, German Primate Center, Göttingen, Germany

^7^ Laboratoire de Neurosciences Cognitives et Adaptatives, UMR 7364, Strasbourg University, France

***Corresponding author**: Fabia M. Miss, Department of Anthropology, University of Zurich, 8057, Zurich, Switzerland. Email: fabia.miss@uzh.ch. ORCID 0000-0002-8971-4477

**This file includes:**

Supplementary Text: Hierarchy analyses in the Tonkean macaques

Supplementary Table S1 – S3

Supplementary Fig. S1 – S4

**Hierarchy analyses in the Tonkean macaques**

We calculated Elo-ratings with the package “EloRating” from a sequence of dominance interactions recorded during two time periods (before and after the joint Simon task experiment) lasting from March 14 until July 5, 2018 and from November 13 until December 13, 2018. In the calculation, we used a startvalue of 1000 and optimized the k parameter. From the interaction matrix, we further calculated David’s scores and normalized David’s scores (Table S1). A Percolation and Conductance (P&C) analysis using the package “Perc” resulted in a similar rank order with minor differences occurring mostly among the intermediate ranks (Table S2). In order to score conflicts in a tolerant macaque population often involving retaliation, we distinguished between these three outcomes:

| A attacks B and A wins | A-B as one winner-loser entry in the conflict matrix |
| --- | --- |
| A attacks B and B retaliates with no clear winner | A-B and B-A as two independent winner-loser entries in the conflict matrix |
| A attacks B and B retaliates and after the fight A wins | A-B and B-A and A-B as three independent winner-loser entries in the conflict matrix |

**Table S1** Overview of Elo-ratings, David’s scores and **Table S2** Rank order based on the P&C method.

normalized David’s scores.

| **Rank** | **ID** | **Elo rating** | **DS** | **normDS** |  | **Rank** | **ID** | **Rank stability** |
| --- | --- | --- | --- | --- | --- | --- | --- | --- |
| 1 | Walt | 1419 | 81.02 | 14.18 |  | 1 | Wallace | 0.88 (0.12) |
| 2 | Wallace | 1346 | 88.79 | 14.54 |  | 2 | Walt | 0.86 (0.14) |
| 3 | Nereis | 1184 | 42.85 | 12.45 |  | 3 | Wotan | 0.72 (0.15) |
| 4 | Olli | 1122 | 30.88 | 11.90 |  | 4 | Ulysse | 0.76 (0.15) |
| 5 | Yang | 1113 | 11.71 | 11.03 |  | 5 | Nereis | 0.79 (0.16) |
| 6 | Ulysse | 1093 | 41.01 | 12.36 |  | 6 | Olli | 0.84 (0.17) |
| 7 | Wotan | 1081 | 19.91 | 11.40 |  | 7 | Yin | 0.78 (0.18) |
| 8 | Yin | 1077 | 20.04 | 11.41 |  | 8 | Olga | 0.78 (0.17) |
| 9 | Patsy | 1072 | 8.47 | 10.89 |  | 9 | Yang | 0.75 (0.18) |
| 10 | Nema | 1028 | 4.17 | 10.69 |  | 10 | Patsy | 0.72 (0.17) |
| 11 | Yannick | 1016 | 3.67 | 10.67 |  | 11 | Yannick | 0.74 (0.18) |
| 12 | Olaf | 989 | -2.04 | 10.41 |  | 12 | Olaf | 0.82 (0.14) |
| 13 | Lady | 962 | -17.75 | 9.69 |  | 13 | Anubis | 0.73 (0.17) |
| 14 | Patchouli | 957 | -16.69 | 9.74 |  | 14 | Nema | 0.81 (0.13) |
| 15 | Anubis | 933 | -3.20 | 10.35 |  | 15 | Abricot | 0.75 (0.18) |
| 16 | Olga | 927 | 7.63 | 10.85 |  | 16 | Patchouli | 0.79 (0.17) |
| 17 | Abricot | 892 | -37.52 | 8.79 |  | 17 | Jeanne | 0.71 (0.16) |
| 18 | Jeanne | 855 | -17.35 | 9.71 |  | 18 | Alaric | 0.80 (0.14) |
| 19 | Alvin | 797 | -47.81 | 8.33 |  | 19 | Lady | 0.80 (0.12) |
| 20 | Yoh | 753 | -80.11 | 6.86 |  | 20 | Alvin | 0.82 (0.13) |
| 21 | Alaric | 747 | -47.48 | 8.34 |  | 21 | Yoh | 0.83 (0.14) |
| 22 | Lassa | 637 | -90.17 | 6.40 |  | 22 | Lassa | 0.83 (0.15) |

**Notes.** Individuals that participated in the tasks are shaded.

**Table S3** Species differences in motor inhibitory control. Output of the binomial glmm with the effect of species, session, age, and sex on inhibitory control assessed with a detour-reaching task (correct or incorrect inhibition trial at first attempt). N = 17 individuals, χ^2^_8_ = 21.48, p = 0.006, ∆AIC = 5.48; R^2^_GLMM_ = 0.68. Significant effects are indicated with p-values in italics.

| **Fixed factor** | **β** | **SE** | **95 % CI** | **OR** | **z** |  | **p** |
| --- | --- | --- | --- | --- | --- | --- | --- |
| Intercept | 2.3 | 1.37 |  |  |  |  |  |
| Species capuchins vs. marmosets | -4.41 | 1.38 | -7.10, -1.71 | 0.01 | -3.20 |  | *1.36 x 10^-3^ *** |
| Species capuchins vs. Tonkean macaques | -2.85 | 1.10 | -5.01, -0.69 | 0.06 | -2.59 |  | *9.58 x 10^-3^ *** |
| Session |  |  |  |  |  |  |  |
| Session, linear trend | 1.61 | 0.39 | 0.84, 2.38 | 5.00 | 4.10 |  | *4.21 x 10^-5^ **** |
| Session, quadratic trend | 0.03 | 0.36 | -0.68, 0.74 | 1.03 | 0.08 |  | 0.93 |
| Session, cubic trend | -0.12 | 0.36 | -0.83, 0.59 | 0.89 | -0.34 |  | 0.73 |
| Session, quartic trend | -0.05 | 0.36 | -0.75, 0.66 | 0.95 | -0.13 |  | 0.90 |
| Age | -0.14 | 0.14 | -0.41, 0.13 | 0.87 | -1.00 |  | 0.32 |
| Sex | 0.22 | 1.06 | -1.85, 2.30 | 1.25 | 0.21 |  | 0.83 |

**Fig. S1** Sociogram showing the affiliative network of the adult and sub-adult Tonkean macaques. Thicker lines represent stronger bonds based on the observed affiliative interactions (grooming and sitting in contact behavior). Node size represents the eigenvector centrality values. Males are colored in blue and females in pink. Individuals that participated in the study have a dark font and non-participants have a light font.


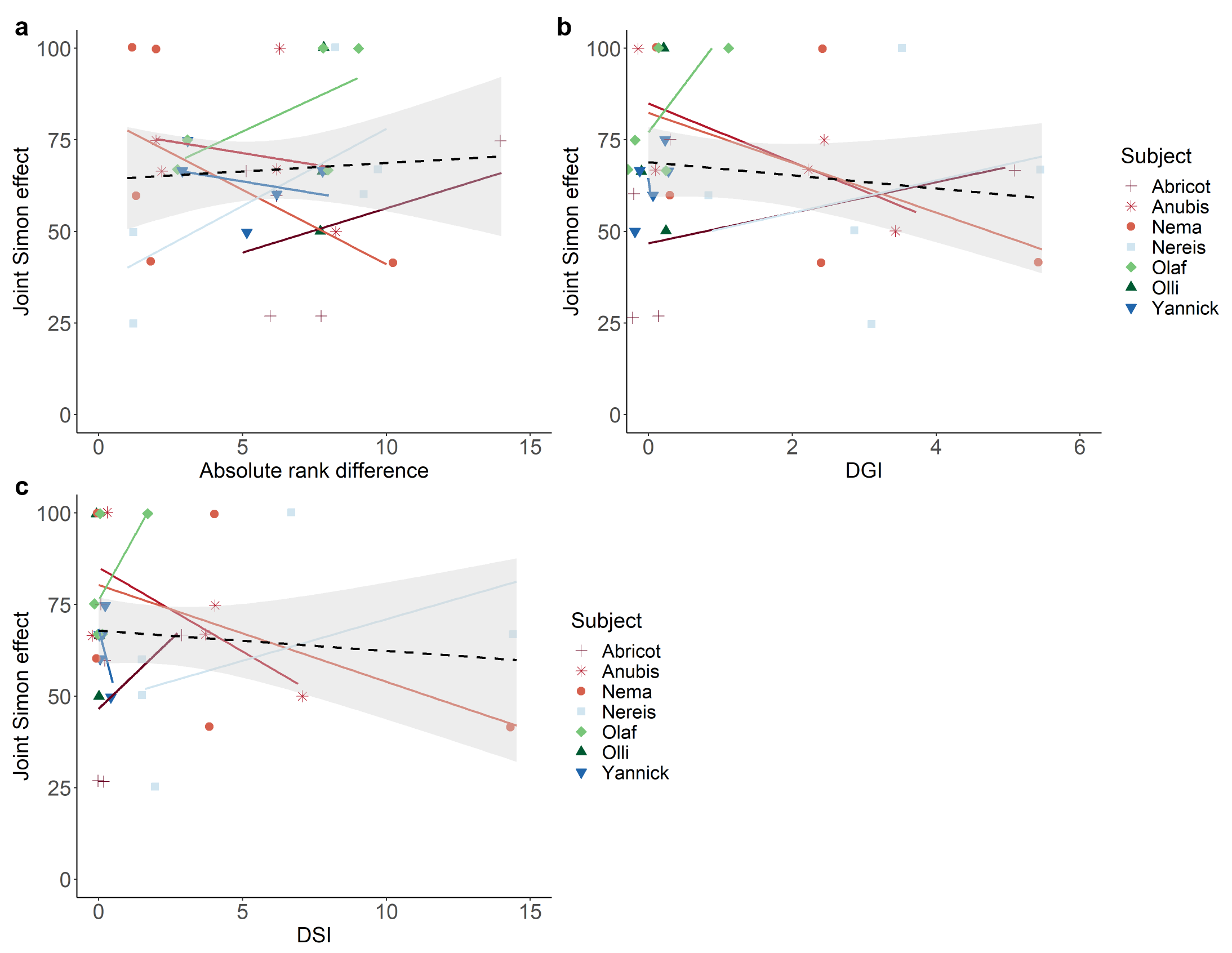


**Fig. S2** The role of social factors in explaining variation in co-representation in the Tonkean macaques. Relationship between an individual’s co-representation with a given partner (i.e., joint Simon effect; % incorrect choices in incompatible minus compatible trials per session) and **(a)** the absolute rank difference, **(b)** the DGI, and **(c)** the DSI (i.e. the bond strength) with the same partner. The regression lines show the correlations between the joint Simon effect and the social factor per individual (solid) and overall (dashed) and do not show model predictions. The shaded areas display 95% confidence intervals. A jitter function was added to better visualize the overlapping data points (absolute rank differences are only integers).

**
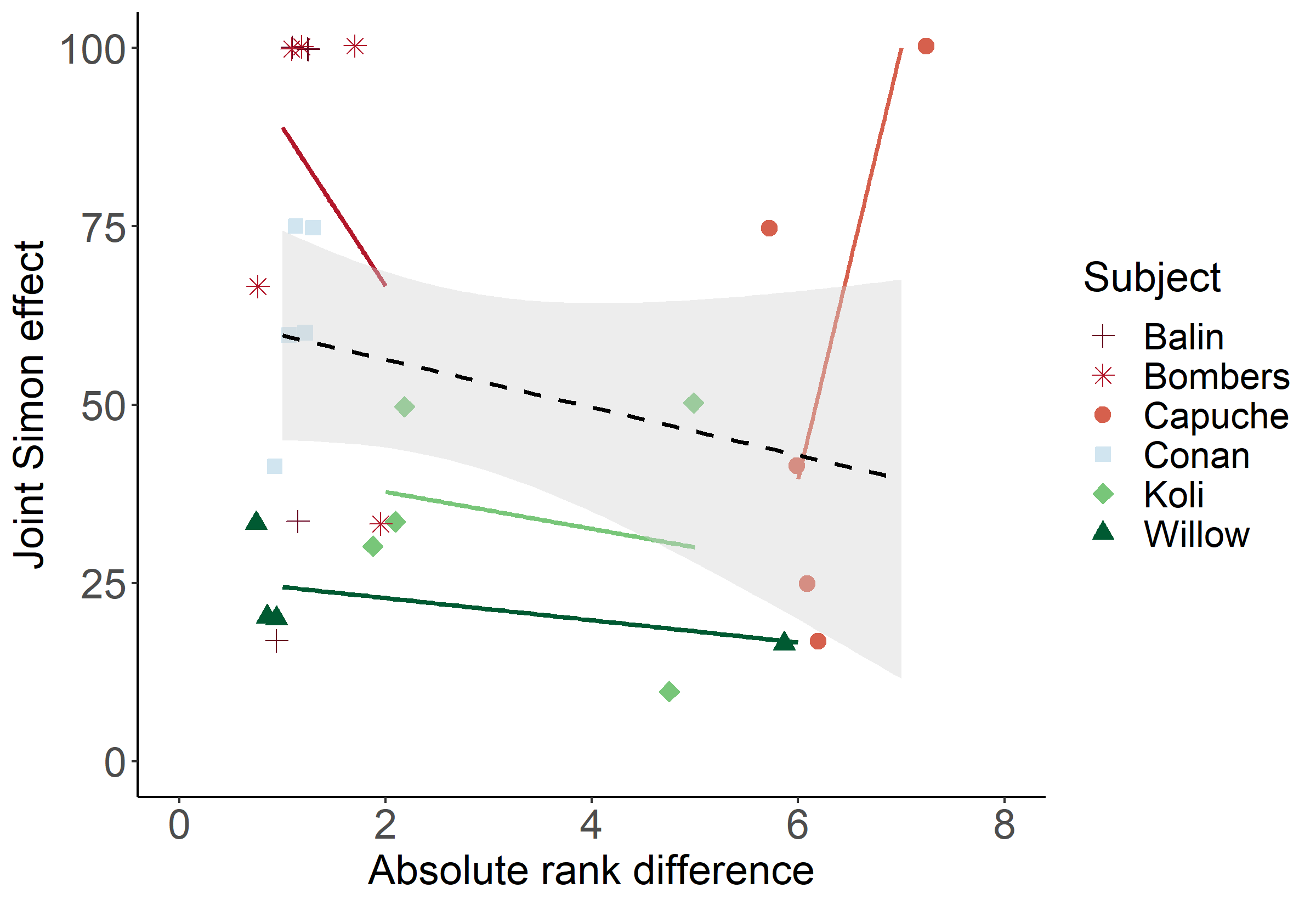
**

**Fig. S3** The role of rank differences between partners in explaining variation in co-representation in the brown capuchins. Relationship between an individual’s co-representation with a given partner (i.e., joint Simon effect; % incorrect choices in incompatible minus compatible trials per session) and the absolute rank difference with the same partner. The regression lines show the correlations between the joint Simon effect and the rank difference per individual (solid) and overall (dashed) and do not show model predictions. The shaded areas display 95% confidence intervals. A jitter function was added to better visualize the overlapping data points (absolute rank differences are only integers).


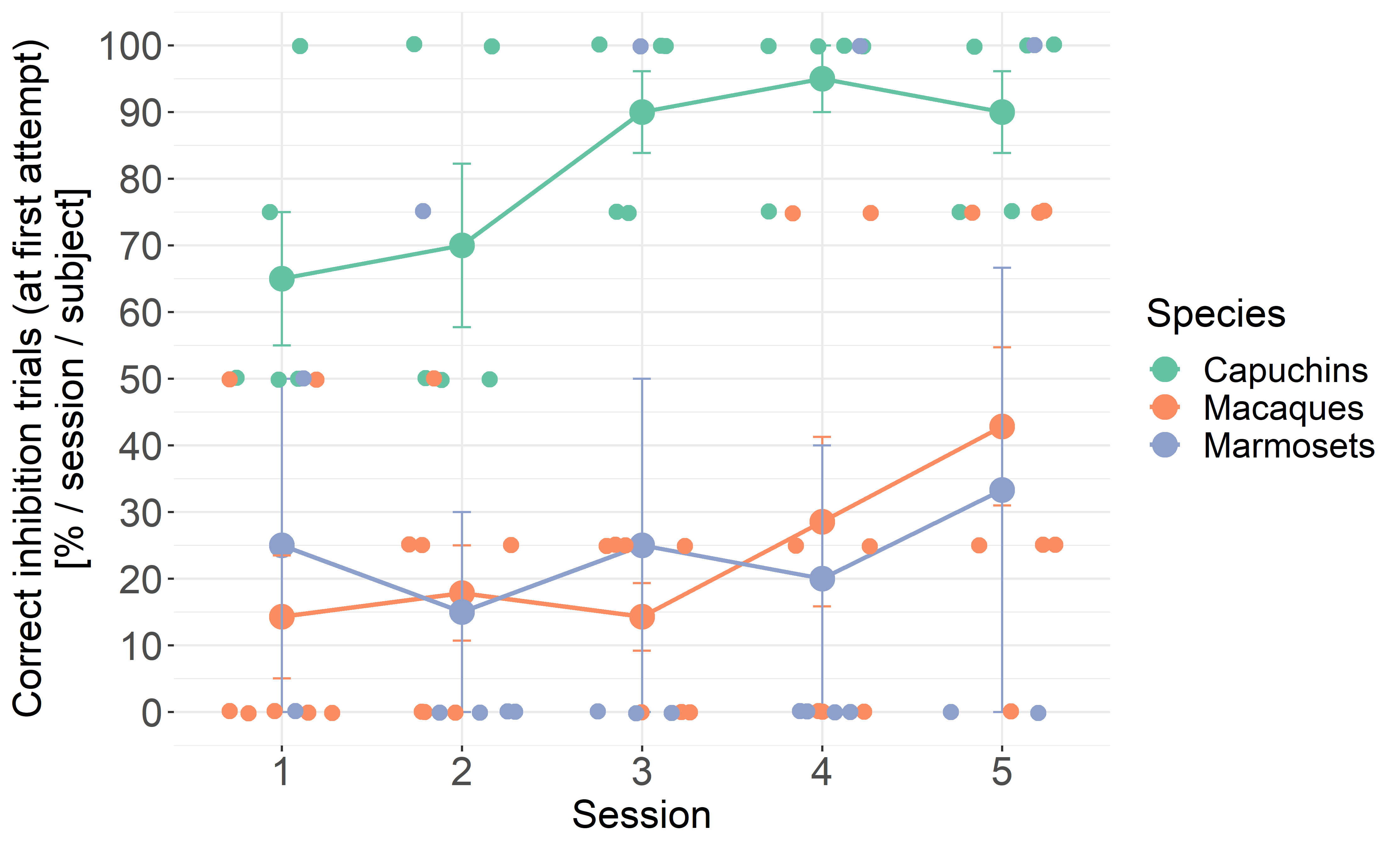


**Fig. S4** Linear trend of session in motor inhibitory control ability. Changes in the individuals’ motor inhibitory control measures (i.e., observed correct inhibition trials at first attempt in the detour-reaching task) over time from session one to five, split up by species. Error bars represent standard errors of the mean. A jitter function was added to better visualize the overlapping data points (session numbers are only integers).
